# Differential Mitochondrial Toxicity Screening and Multi-Parametric Data Analysis

**DOI:** 10.1371/journal.pone.0045226

**Published:** 2012-10-15

**Authors:** Maria V. Tsiper, Jennifer Sturgis, Larisa V. Avramova, Shilpa Parakh, Raymond Fatig, Ana Juan-García, Nianyu Li, Bartek Rajwa, Padma Narayanan, C. W. Qualls, J. Paul Robinson, V. Jo Davisson

**Affiliations:** 1 Bindley Bioscience Center at Purdue University Discovery Park, West Lafayette, Indiana, United States of America; 2 Department of Medicinal Chemistry and Molecular Pharmacology, Purdue University, West Lafayette, Indiana, United States of America; 3 Department of Basic Medical Sciences, Purdue University, West Lafayette, Indiana, United States of America; 4 Weldon School of Biomedical Engineering, Purdue University, West Lafayette, Indiana, United States of America; 5 Comparative Biology and Safety Sciences, Amgen Inc, Seattle, Washington, United States of America; Duke University, United States of America

## Abstract

Early evaluation of new drug entities for their potential to cause mitochondrial dysfunction is becoming an important task for drug development. Multi-parametric high-content screening (mp-HCS) of mitochondrial toxicity holds promise as a lead *in-vitro* strategy for drug testing and safety evaluations. In this study, we have developed a mp-HCS and multi-parametric data analysis scheme for assessing cell responses to induced mitochondrial perturbation. The mp-HCS measurements are shown to be robust enough to allow for quantitative comparison of biological systems with different metabolic pathways simulated by alteration of growth media. Substitution of medium glucose for galactose sensitized cells to drug action and revealed novel response parameters. Each compound was quantitatively characterized according to induced phenotypic changes of cell morphology and functionality measured by fluorescent biomarkers for mitochondrial activity, plasma membrane permeability, and nuclear morphology. Descriptors of drug effects were established by generation of a SCRIT (Specialized-Cell-Response-to-Induced-Toxicity) vector, consisting of normalized statistical measures of each parameter at each dose and growth condition. The dimensionality of SCRIT vectors depends on the number of parameters chosen, which in turn depends on the hypothesis being tested. Specifically, incorporation of three parameters of response into SCRIT vectors enabled clustering of 84 training compounds with known pharmacological and toxicological activities according to the degree of toxicity and mitochondrial involvement. Inclusion of 6 parameters enabled the resolution of more subtle differences between compounds within a common therapeutic class; scoring enabled a ranking of statins in direct agreement with clinical outcomes. Comparison of drug-induced changes required variations in glucose for separation of mitochondrial dysfunction from other types of cytotoxicity. These results also demonstrate that the number of drugs in a training set, the choice of parameters used in analysis, and statistical measures are fundamental for specific hypothesis testing and assessment of quantitative phenotypic differences.

## Introduction

A growing number of diseases, including diabetes, cardiovascular diseases, cancers and neurodegenerative processes have been linked to mitochondrial dysfunction [1], [2]. An emerging understanding of the roles of the mitochondria in the overall integration of cellular signaling events motivates the creation of new methods and tools to understand the behavior of systems in the context of mitochondrial functions [3], [4]. Additional meaningful insights linking molecular changes to physiological effects motivate more integrated approaches to understanding phenotypic fate of cells and organs. Among these objectives is the use of *in vitro* higher content phenotypic assays that have potential to shed insights on the role of specific molecular events in all cell and population response.

There are established arenas where mitochondrial dysfunctions are important for recognition and quantification of early apoptotic commitment. In particular, drug-induced damages to cardiac and liver tissues are common causes of therapy related organ failures. The primary side effects of various drugs have been attributed to mitochondrial toxicity resulting in promising drugs being withdrawn from the market [5]. Often, timely detection of undesired toxicity enables the development of a safer therapy which can even include considerations of pharmacological protection of sensitive tissues [6]. An example of this approach is the use of Dexrazoxane cardio-protection from anthracycline-induced toxicity [7]. Early-stage quantitative assessment of tissue-specific, drug-induced mitochondrial toxicity is a clear need for drug development and safety pharmacology. However, there continues to be a deficiency in the platform-independent metrics to classify all drugs for potential clinical risk.

The mitochondrial membrane potential (MMP) is critical in maintaining the driving force for oxidative-phosphorylation and ATP synthesis through ADP phosphorylation by mitochondrial ATP synthase (complex V). Changes in MMP often reflect mitochondrial activity and cellular commitment to apoptosis. In response to pro-apoptotic signaling factors or ROS generation, mitochondrial membrane permeability changes, causing the characteristic drop in MMP and further release of pro-apoptotic factors [8]. In addition to the intrinsic apoptotic pathway, other processes impact the MMP including necrotic cell death and non-apoptotic mitochondrial uncoupling. Whether MMP loss is an instrumental step of the apoptotic pathway, an epi-phenomenon of cell death, or a manifestation of other mitochondrial mechanisms often remains inconclusive. There are several general mechanisms associated with known drug-induced MMP changes, including (i) inhibition of mitochondrial complexes I-V and ANT (adenosine nuclear transferase), (ii) mitochondrial uncoupling, (iii) activation of pro-apoptotic signal transduction pathways that lead to the formation of mitochondrial membrane permeability pores, and (iv) induction of non-mitochondrial cell death accompanied by a drop in MMP at a late stage [5], [9]. Distinguishing between these or other molecular mechanisms is a highly significant but challenging problem which solution will enable to predict clinical risk of new or investigational drugs.

Cellular regulation of mitochondria-mediated ATP generation and apoptosis, along with glycolytic ATP generation enables cells to cope with various nutrition environments and manage diverse energy demands. Substitution of galactose for glucose in the cell culture medium proved to inhibit or significantly downregulate net cytoplasmic ATP production and leave mitochondrial oxidative phosporylation as a major mechanism of ATP synthesis, thus sensitizing cells to mitochondrial abnormalities [10], [11]. Evaluation of mitochondrial dysfunctions using comparative drug effects between glucose and galactose carbohydrate sources in cell culture medium of HepG2 and H9c2 cells demonstrated sensitization of galactose-feed cells to mitochondrial toxicants [4], [12]. However, the analysis of cell response under different medium conditions was limited to the visual comparison of dose-response curves or comparison of IC_50_s, rendering it a system with limited content.

Development of robust multi-parametric high content screen (mp-HCS) which enables automated assessment of mitochondrial perturbations based on the differential cell responses to compounds in two different growth conditions is a new approach. We implemented imaging cytometry techniques to simultaneously monitor several parameters extracted from quantitative analysis of biomarker intensity and distribution, including MMP (TMRM), plasma membrane permeability (TO-PRO-3), and nuclear morphology (Hoechst33342), as functions of compound concentration and medium carbohydrate content. A training library of 84 compounds was assembled to include compounds with variable degrees of chemical similarity and expected mechanisms of action, including mitochondrial respiratory chain inhibitors, oxidative stress inducers, DNA disrupting agents, and kinase inhibitors. The basis of our differential mitochondrial toxicity mp-HCS is the assumption that drug-induced activation of pro-apoptotic signaling will not be affected by the decrease of glycolytic ATP. However, drugs that cause direct perturbations to mitochondrial machinery will have detrimental effects on cellular viability under conditions when glycolysis is not available. Simultaneous analysis of multiple cellular features at identical compound doses but in different culturing conditions provided data to develop a schema for automatic separation of mechanistic classes through the application of multivariate analysis (multiparametric image cytometry).

Common approaches for evaluation of multiparametric response in HCS and cytometry involve identification of the earliest responsive parameter [4], [13], [14], [15], and correlation analysis of measured parameters with or without factor analysis [16], [17], [18]. A hypothesis-driven selection of conditions, parameters, and statistical distance measurements was pursued as an approach toward mining of information-rich data sets. Specifically, a data-driven statistical model for assessing differential responses to induced mitochondrial toxicity has been developed. The essence of differential response analysis is that in addition to characterization of cell response to a compound, the change of cell responses as a function of environmental conditions are analyzed leading in essence to a multifactorial response dataset. The data reduction involves the construction of an n-dimensional specialized cell response to induced toxicity (SCRIT) vector consisting of a number of normalized statistical measures of pre-defined parameters. A single SCRIT vector characterizing multi-parametric cell response to compound action was composed for each drug. Correlation analysis of SCRIT vectors enabled automatic clustering of compounds into five predicted categories according to the induced toxicity response: direct inhibition of mitochondrial machinery, glucose-independent induction of pro-apoptotic signaling, glucose-dependent toxicity, mitochondria uncoupling, and no toxicity response. Moreover, an increase in the number of parameters of cell response enabled further subdivision of compounds into more narrow clusters to appreciate fine differences in phenotypic cellular response to a specific compound.

## Results

### Assay development and parameter definitions

The experimental design involves comparative analysis of differential drug effects in two biological settings with ultimate normalization to controls. This requirement places demands on understanding experimental sources and precision in estimates of error. Briefly, assay development included optimization of the seeding cell density to achieve maximal cell count and accurate Hoechst33342–based nuclear segmentation using the iCys software, 48 h after plating. A seeding concentration of 4×10^4^ cells/well was established to be optimal for HepG2 cells under in-house culturing conditions (see Materials and Methods and Methods S1). Cells were seeded 18 h to 24 h before the addition of compounds. After 24 h of cell exposure to a compound, the cell marker cocktail was added to the medium for 45 min. Cultures were then transferred to the iCys imaging cytometer for data collection. The cell-permeable nuclear marker Hoechst33342 was used for nuclear segmentation and cytoplasmic or peripheral area definition as well as for nuclear morphology characterization. TMRM, a lipophilic fluorescent cationic dye marker, was used to monitor MMP changes [19]. TO-PRO-3 membrane-impermeable nuclear marker was used to characterize plasma membrane integrity and, hence, cell viability.

The protonophore FCCP (protonophore trifluoromethoxy carbonyl cyanide phenylhydrazone) was chosen as the end-point control for assay optimization for both MMP change (TMRM signal decrease) and cell death (TO-PRO-3 signal increase). This compound renders the mitochondrial membrane permeable to protons and results in immediate MMP dissipation and uncoupling of oxidative phosphorylation from ATP synthesis. Assay parameters and conditions were established and experimental error was estimated using multiple repeats of untreated and FCCP-treated samples. Specifically, assay development utilized a 96-well plate containing 6 columns treated with 75 µM FCCP (48 FCCP-treated samples) and 6 columns treated with 0.2% DMSO (48 untreated samples) for 24 hours. The plates were used for (1) definition of statistical measure of parameters, (2) well-to-well variability calculation, and (3) estimation of Z′ showing separation between positive and negative controls, (4) variability between multiparametric vectors assessed using correlation analysis.

**Figure 1:**
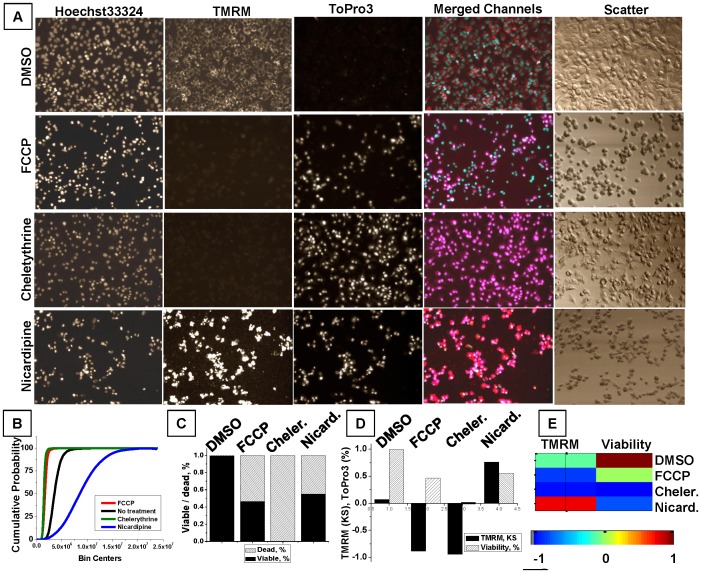
Examples of HepG2 response to treatment with different compounds with various types of quantitative analysis and data representation. Hoechst33342, TO-PRO-3, and TMRM were used to characterize the untreated HepG2 population and the cell toxicity response induced by different compounds. Cells were seeded at the same time and density, incubated for 24 h with 100 µM of different compounds, stained with a cell marker cocktail, and analyzed using an iCys imaging cytometer. **A.** Representative images (500×368 µm) demonstrate fluorescent signal distribution for individual channels. A merged color image representing a combination of channels (TMRM-red, Hoechst33342-blue, TO-PRO-3-magenta) is shown to demonstrate co-localization. A light-scatter image is shown to realize cell morphology. **B.** Cumulative probability functions demonstrate TMRM change induced by different treatments: FCCP - red line, chelerythrine – green line, nicardipine – blue line, and untreated (DMSO) - black line. **C.** Bar graph represents the fraction of viable (TO-PRO-3-negative) cells (black), and the fraction of dead (TO-PRO-3-positive) cells (shaded). **D.** The bar graph represents responses of two parameters, TMRM KS values (solid bars) and the fraction of viable cells (shaded), to various treatments. **E.** Color-coded representation of 2-parameter response for each of four compounds. Viability is rescaled from −1 (all dead) to +1 (all live) to match the range for KS values.

Typical fields of view for untreated and FCCP-treated cells are shown in Figure 1A. FCCP-treated cells exhibit dramatic change of cellular morphology: cells become round and retain no cytoplasmic spreading, based upon manual examination of light-scatter images. Changes in nuclear morphology are equally drastic, with nuclei becoming rounded and reduced in size, with condensed nuclear material (Hoechst33342 channel). Dissipation of MMP is visualized by the loss of cytoplasmic, perinuclear, mitochondria-specific TMRM distribution. Finally, FCCP-treated cells contained two cell populations, TO-PRO-3-positive and TO-PRO-3-negative. A population of TO-PRO-3-positive cells corresponds to late apoptotic or necrotic cells (TO-PRO-3 channel), while a population of TO-PRO-3 negative cells represents cells either at early stage of apoptosis or cells with uncoupled (inactive) mitochondria since mitochondrial uncoupling by FCCP does not necessarily lead to apoptosis [20].

**Figure 2:**
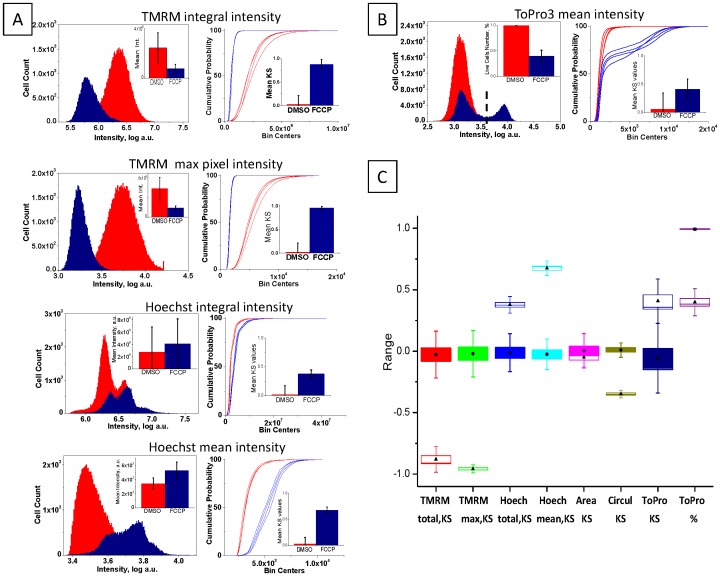
Definition and selection of statistical measure of parameters. Forty-eight FCCP-treated and 48 non-treated (DMSO) samples were used for statistical analysis of population variability. Seven iCys output parameters were selected for the analysis: TMRM peripheral integral and max pixel intensity, Hoechst33342 integral and mean intensity, nuclear area and circularity, and TO-PRO-3 mean intensity. **A.** Right panel shows histograms of parameter distribution for untreated (red) and FCCP-treated (blue) samples. Insert bar graphs demonstrates mean values with standard deviation for all parameters. The left panel shows the corresponding cumulative probability functions (CPFs), which are the basis for the Kolmogorov-Smirnov (KS) value calculation. Representative four repeats of untreated (blue lines) and four repeats of FCCP-treated (red lines) samples are individually plotted to demonstrate the deviation and robustness of each parameter. Insert bar graph demonstrates mean KS values with standard deviation calculated using averaged untreated population. **B.** Right panel shows histograms of parameter distribution for untreated (red) and FCCP-treated (blue) samples. An empirical gating value (punctuate line) was used to quantify the number of TO-PRO-3-positive cells; and % live (TO-PRO-3-negative) cells is plotted in the insert. The left panel shows the corresponding CPFs for four representative repeats of untreated (blue lines) and FCCP-treated (red lines) samples. Insert bar graph demonstrates mean KS values with standard deviation calculated using averaged untreated population. **C.** Modified box-and-whiskers graph demonstrates comparative behavior of 8 statistical measures: 7 parameters as KS values and a TO-PRO-3-derived viability index in %. Boxes correspond to 95% confidence interval of the mean calculated based on 48 repeats (black circles - mean values for untreated; black triangles - for FCCP-treated populations). Standard deviation of the mean is also shown. Red - TMRM peripheral integral intensity, green - max pixel intensity, dark blue - Hoechst33342 integral intensity, blue - mean intensity, magenta - nuclear area, yellow - nuclear circularity, dark purple - TO-PRO-3 mean intensity, light purple - viability in %.

To quantify cell responses to FCCP, seven measured parameters were analyzed and compared between treated and untreated populations. Histograms of the population distribution of selected parameters are shown in Figure 2. A small but significant (p≪0.05) difference between arithmetic means was demonstrated for normally distributed parameters. The bar graphs (Figure 2A, left inserts) demonstrate mean and standard deviation calculated based on 48 FCCP-treated and 48 untreated samples. However, for non-normal distributions, and moreover, bi-modal distributions of responses, Kolmogorov-Smirnov (KS) distance was implemented as a dissimilarity measure normalized against the in-plate untreated controls. KS distance has been previously used for measuring dissimilarity of distributions in high-content image data analysis [15], [16]. In this study, this distance is defined as the maximum signed difference between cumulative distribution functions (CPFs) which were built for each of the 48 FCCP-treated samples and the 48 untreated controls. Four representative cumulative probability functions are plotted independently for each parameter for both treated and untreated conditions to demonstrate the high robustness (Figure 2A right); KS mean values and standard deviations are plotted as a bar graph (Figure 2A, right inserts).

The type of distributions of values measured for individual parameters are in principle unknown. For this reason, each parameter was individually analyzed and the best statistical measure representing dissimilarity between populations was selected. While gradual concentration-dependent changes of TMRM and Hoechst33342 intensity represent the biological measure of MMP and nuclear changes, for TO-PRO-3 only the end-point responses meaningfully reflect the plasma membrane status. An empirical gating approach to quantify TO-PRO-3-positive and -negative cell populations was employed (Figure 2B, left graph); the statistical measure of viability represents a percent of positive cells (viability factor). A bar graph demonstrates the mean viability with standard deviation calculated based on 48 untreated and 48 FCCP-treated samples (Figure 2B left graph, insert). Note that about 50% of FCCP-treated cells remain viable (or TO-PRO-3-negative). The CDF of TO-PRO-3 mean intensity (Figure 2B, left graph) reflects the bimodal nature of the TO-PRO-3 distribution and demonstrates high variability when KS distance is used (Figure 2B, left graph insert). The TO-PRO-3 empirical gating values were determined for each plate according to visual examination of TO-PRO-3 distribution. For all analyzed plates, empirical TO-PRO-3 gating values were between 1.8×10^3^ and 2.5×10^3^. Figure 2B summarizes the behavior of dissimilarity measures for 7 parameters, including 2 measures of TO-PRO-3 response for comparison (KS and percent viability). The modified box-and-whisker graphs demonstrate confidence intervals of the mean and standard deviation for different measures of response (Figure 2C).

A single parameter (with the exception of nuclear area) proved to be sufficient to distinguish between untreated and FCCP-treated populations based on Wilk's lambda test (data not shown). However, in order to distinguish other phenotypic categories more than one image-based parameter must be included. For example, two parameters characterizing MMP (KS of TMRM peripheral integral intensity) and viability (% of TO-PRO-3-negative cells) enabled us to distinguish 4 visually appreciated phenotypic categories: (1) no change of MMP and viability, (2) partial response, as MMP decrease with partial loss of viability, (3) complete response, as MMP and viability drop, and (4) unexpected response, as MMP increase but viability decrease (Figure 1).

To differentiate the cellular responses to FCCP, chelerythrine, nicardipine, and DMSO under two environmental conditions, TMRM KS and viability were sufficient parameters for quantification of these treatments (Figure 1B–E). Treatment with FCCP resulted in a drop in MMP accompanied by some decrease in viability. In contrast, treatment with chelerythrine, a potent protein kinase C inhibitor, resulted in 100% MMP and viability loss. Nicardipine, a calcium-channel blocking agent, at high concentration (100 µM) caused a surprising increase of TMRM, accompanied by a decrease in cell viability. Image analysis revealed no increase in background TMRM channel fluorescence, whereas cell-associated TMRM staining was greatly increased even for TO-PRO-3-positive cells (Figure 1A). A graph of CPFs for TMRM intensity reflects the visually-appreciated TMRM signal increase for nicardipine as a shift to the right from the DMSO-treated control sample (Figure 1B, blue line) and positive sign of KS value (Figure 1D–E), while FCCP- and chelerythrine-induced TMRM decrease is reflected by a left shift of CPFs (Figure 1B, red and green lines) and negative sign of KS value (Figure 1D–E).

### Differential dose response and SCRIT vector construction

After the end-point assay optimization and parameter definition, analysis of 10-point dose-response screens executed in quadruplicate for known mitochondria toxicants FCCP, ionomycin, rotenone, and antimycin A was performed. FCCP-treated cells exhibited gradual loss of MMP accompanied by a similar decrease in viability (Figure S3A, first and second graphs). Interestingly, strong mitochondrial respiratory complexes inhibitors rotenone and antimycin A demonstrated modest or no effect on MMP and viability, respectively (Figure S3A, left two graphs).

**Figure 3:**
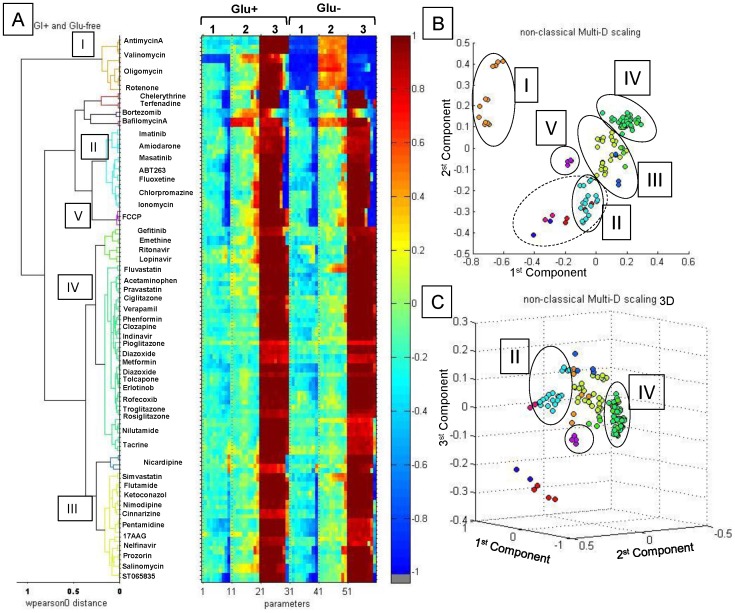
Correlation analysis of SCRIT vectors. Correlation analysis was performed using pair-wise Pierson distances of SCRIT vectors. **A.** Dendogram of hierarchical clustering of SCRIT vectors in the 40-dimensional parameter space incorporating glu+ and glu− data points. Color scale is shown on the right. **B.** 3D plot of MDS of SCRIT vector correlation analysis. **C.** 2D plot of MDS of SCRIT vector correlation analysis: I - direct mitochondrial toxins, II - glu-independent toxins, III - glu-dependent toxins, IV - non-toxic or inactive compounds, and V - uncoupling compounds such as FCCP.

The same compounds were analyzed in glucose-free (glu−) medium conditions, applying a nutrient-sensitization strategy to shift cellular metabolism from cytoplasmic glycolysis to mitochondrial oxidative phosphorylation [10]. HepG2 cultures grown in medium with galactose as the major carbohydrate source demonstrated increased sensitivity to mitochondrial toxins, especially to inhibitors of oxidative phosphorylation machinery (Figure 3A, right two graphs). MMP and cell viability of cells treated with rotenone and antimycin A showed significant decreases after substitution of galactose for glucose. The TMRM dose response to FCCP demonstrated little or no change with a striking decrease in viability. Intriguingly, dose responses of ionomycin did not change in different culturing media.

The variation in cell responses to compound treatments depending on glucose availability is best illustrated when two parameters, TMRM KS based upon peripheral integral signal (PI) and the viability index, for two nutrient conditions (glu+ and glu− media) are plotted (Figure S3B). Antimycin A and rotenone are characterized by a similar trend of responses as a dramatic decrease of both TMRM and viability in glu− conditions. Cell response to ionomycin reflects no change of toxicity between glu+ and glu− conditions. A completely different pattern of cell response to FCCP is revealed by a change in viability along with little change in TMRM. Heat-map representation of dose response for KS values of TMRM signal and viability indexes for glu+ and glu− media conditions (Figure S3C) enabled more compact data visualization. Differential multi-parametric cell responses to a specific compound are referred to as specialized-cell response-to-induced-toxicity (SCRIT) vectors. In this example, 40-point SCRIT vectors were constructed from two parameters of cell response (MMP PI and cell viability), 10 concentrations (from 0.005 µM to 100 µM), and two nutrient medium conditions (glu+ and glu−) for each repeat of each compound. Other parameters defined in Figure 2 were added to SCRIT vectors when necessary for a specific analysis.

### Primary screening and compound selection

We performed a pre-screening using a single high dose (100 µM) of each drug from a library of 74 (excluding 10 control toxicants). Plate layout for this screen is displayed in the Supporting Information (Figure S4). Briefly, the last column of a 96-well plate contained four positive controls (FCCP at 75 µM) and four DMSO-treated negative controls; other wells were treated with different compounds. Duplicate plates for each carbohydrate condition (glu+ and glu−) were prepared. Images were visually analyzed for toxicity response and staining abnormalities after 24 h incubation of cells with compounds. Euclidean distance was used for multidimensional scaling and hierarchical clustering to visualize and exam SCRIT vectors in order to select responsive compounds for secondary screening. Non-active compounds were designated as group IV compounds (Table S1). In the primary screens, an arbitrary cut-off of 20% change in any of the seven values composing SCRIT vectors was used to select active compounds. This cut-off value is safely above coefficients of variation for the repeated controls. The entire process was manually verified by inspection of morphological changes visible in transmitted light iCys images; selection of compounds like Nilutamide which was incorrectly assigned to Group IV on the primary screen were identified. Several compounds were found to be incompatible with the live-cell-no-wash protocol and automated LSC analysis due to autofluorescence or interference with Hoechst33342 nuclear staining and impairment of nuclear segmentation (Figure S5). These compounds were removed from the subsequent automated analysis but the data obtained are available for visual examination and interpretation.

### Automated SCRIT analysis and compound clustering

Secondary screening was performed using the pre-selected set of 53 compounds, which included a group of well-characterized mitochondrial toxins, a group of compounds that demonstrated phenotypes of response in primary screening, and chemical or pharmacological analogs of responsive compounds. Data from 10-point dose response experiments were collected in duplicate or quadruplicate and SCRIT vectors were built for each repeat independently using data from both medium conditions. The final 96-well plate layout for the secondary (dose response) screening contained two repeats of ten, 1∶3 dilutions of each compound, along with eight positive (treated with 75 µM FCCP), and eight negative (DMSO-treated) controls.

Further data reduction was performed by multidimensional scaling (MDS) and hierarchical clustering of SCRIT vectors for each of the 10 step dose replicates in two medium conditions. Abbreviated SCRIT vectors based upon three parameters with highest discriminatory power derived from individual cellular markers were used: KS distance between distributions of TMRM PI, KS distance between Hoechst33342 mean cell intensity, and percentage of viable cells or viability (Figure 3). For the purpose of multidimensional scaling and hierarchical clustering, the similarity (distance) between compounds was defined as Pearson distance between SCRIT vectors. The dendrogram in Figure 3A reflects groups of compounds with similar biological responses; smaller distances reflect larger similarity between compounds. Replicates of each compound were analyzed independently and the corresponding replicate data formed the clusters with the smallest pair-wise comparison distances, demonstrating consistent reproducibility of the assay and analysis (dissimilarity less than 0.2). Compounds which demonstrated high pair-wise dissimilarity of their repeats (>0.2) were removed from analysis as outliers, for example amitriptylene and haloperidol. Multidimensional scaling was performed to visualize the similarities between different compounds (Figure 3B, C) in two dimensional formats. Mitochondrial toxins with direct action such as antimycin A, oligomycin, valinomycin, and rotenone formed one well-defined cluster I (Figure 3, orange). This cluster is marked as I on the clustering dendrogram and 2D graph of MDS analysis. Dramatic decreases in MMP and viability and increase in nuclear intensity are characteristics for compounds in this cluster. Valinomycin is assigned to cluster I despite no change in TMRM behavior between glu− and glu+ conditions possibly because its dose response is not complete due to MMP dissipation even at the lowest tested concentration (5 nM).

Compounds in cluster II (Figure 3, blue) displayed significant cytotoxicity in glucose-independent fashions. Another group (cluster III) includes compounds that demonstrate mitochondrial perturbations only under the sensitizing conditions with prominent changes of parameters after glucose withdrawal (Figure 3, yellow). A large group of non-toxic or inactive compounds are combined in cluster IV (Figure 3, red). Several small groups include (a) a cluster of FCCP repeats that demonstrate a dramatic change in viability with little or no change in TMRM dose response upon glucose withdrawal; (b) a cluster formed by nicardipine, which caused a spectacular increase in TMRM along with a drop in viability in both medium conditions; (c) bortezomib, bafilomycin A, terfenadine, and chelerythrine demonstrated a similar response in both conditions and hence could be assigned to group II (Figure 3B, punctuate line). The terfenadine SCRIT revealed an artifact in TO-PRO-3-positive cells at the highest concentration; manual examination of images ultimately re-assigned this compound to group II.

## Discussion

Sensitivity to a compound in the *in-vitro* cell-culture model is a capricious parameter because it depends on many experimental parameters, including passage number, quality of plastic coating, trypsinization procedure, and is sensitive to subtle variations of experimental conditions such as extent of cells adhesion, compound incubation time, or temperature modulations. For example, 24 h of HepG2 incubation with 75 µM FCCP routinely resulted in the presence of a subpopulation of viable cells with greatly decreased MMP. However, this population is sensitive to incubation conditions and might disappear through prolonged exposure to room temperature during scanning. Our results demonstrate feasibility for development of a robust experimental platform to assess compound effects on populations of live cells, and feasibility for creation of a statistical model. Using a set of biomarkers to characterize mitochondrial activity and cell viability, we were able to distinguish between different types of mitochondrial involvement in the response to 84 tested compounds acting via diverse modes. Comparison of changes of dose response collected at uniform conditions decreases the possibility of misleading representation of a single-dose response shift. Cell-population dose responses in different medium conditions were incorporated into a single multi-parametric analysis culminating in semi-automated clustering of drugs.

Parameters such as total, average, or maximal cell marker intensity per cell and area were computed from measured marker fluorescence. The best dissmilarity measure among mean, medium, percent of gated cells, or KS distance between populations was selected for each parameter individually based on the Z′ value calculated for populations of cells treated with FCCP (positive control). The KS distance was established to be the best measure for parameters derived from Hoechst33342 and TMRM in terms of Z′-expressed separation between positive and negative controls, in-plate normalization against untreated control, and uniformity of normalization between different parameters. For TO-PRO-3-derived descriptors the percent of live cells proved to be a better quantitative measure since only the binary “yes” or “no” response was meaningful while gradual changes of TO-PRO-3 were irrelevant for current study. Hoechst33342 is a membrane-permeable nuclear marker whose intensity depends on the nuclear content of the cell; membrane-impermeable TO-PRO-3 is the measure of membrane permeability, and its functional dependence on nuclear content is not well established. The use of KS distance as a dissimilarity measure of image-based biological assays has been previously demonstrated [16]. If other descriptors are added to the analysis, the respective dissimilarity measures should be selected by taking into consideration, distributions of responses and a priori knowledge of cell marker targets. This approach enables construction of response vectors incorporating different measures of dissimilarity for individual cellular responses; a more comprehensive summary descriptor is the result.

A strategy to increase the dimensionality (feature space) to reflect environmental influences on drug action is enabled by the use of different growth conditions. Analysis of dose response curves has become common practice in high-content analysis because it provides a dynamic function of response to drug concentration [21]. However, comparison of dose responses between different environmental conditions is an under-evaluated approach [22]. This N×10×2-dimensions multifactorial response dataset was scaled, control-normalized and finally vectorized to create SCRIT vectors. Besides the increase in feature space, a major advantage of this approach is that differing cell sensitivity to individual compounds is standardized; the analysis compares sensitivities at two different conditions, rather than employing an absolute value of the responses.

The key advantages of the differential cell-response and unsupervised classification platform are: (a) robustness, (b) amenability to automation, and (c) uniformity between parameters and d) potential platform independence. Moreover, the differential screen comparing responses in two environments reduces the impact of incubation time and concentrations on the classification outcome. The absence of pre-conceived parametric model for dose responses also reduces the influences of aberrant effects caused for example by high compound concentrations or iCys incubation times during the data acquisition.

A major problem encountered for the classification assay is the undesired compound-marker interactions occurring for some of the tested chemicals. This is an important but not well recognized problem for all HCS, but specifically for live-cell analysis, which preferentially uses “add-in” protocols where compounds are present in medium at time of analysis [23]. Two important causes of possible false responses are compound autofluorescence and compound interference with the cell marker dyes [24]. Compound autofluorescence can overlap with the emission spectrum of a particular cellular marker and mask or alter the signal; compound emission spectra overlap with Hoechst33342 impedes proper image segmentation. Therefore, a pre-screen image analysis protocol was employed to identify possible interferences with normal marker fluorescence.

The proper choice of image features (extracted cellular parameters) for HCS experiments is a complex image-processing and pattern-recognition problem. Usually the process relies on expertise of investigators or uses algorithms for data-reduction and selection [15], [18]. The approach presented here used a hypothesis-testing rationale where the selection of measured parameters was based on cellular functions assumed to be affected by the compounds.

Monitoring of MMP and cellular viability under conditions of varied glucose content are especially important since glucose withdrawal sensitizes cells to mitochondrial abnormalities [10], [11]. MMP changes may not necessarily be an initial committed step of apoptosis, but may accompany necrotic cell death or even reflect mitochondrial uncoupling. Decrease of cell viability after glucose withdrawal delineates the mitochondrial component of the compound-induced toxicity as the cytoplasmic glycolytic alternative of ATP generation is abolished. In normal medium, inhibition of mitochondrial functions by a compound may be compensated for, and masked by cytoplasmic glycolysis, but revealed under reduced glucose conditions. At the same time, independence of compound action from glucose availability suggests activation of apoptotic signaling pathways that are not compensated by glycolysis.

For the mp-HCS developed here, the employed measure of inter-compound distance can be potentially used to automatically identify similarities between newly tested compounds and the previously tested substances. The broader question addressed in the current study is whether a semi-automated imaging platform can be used to classify MMP-affecting compounds into functional classes: direct inhibitors of mitochondrial complexes, general modulators of mitochondrial function, direct inducers of intrinsic apoptotic pathways, or caused no phenotypic changes.

**Figure 4:**
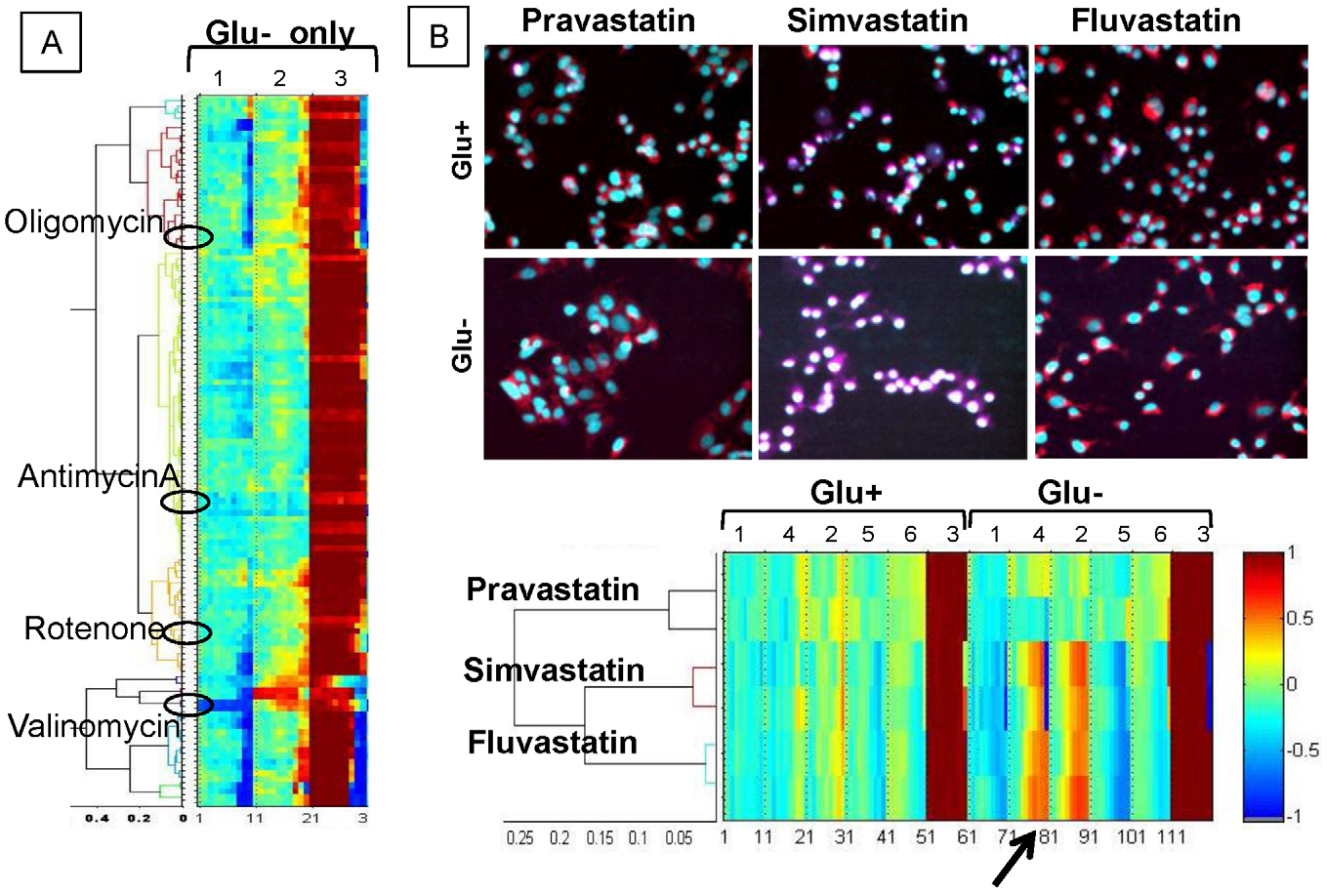
Examples of the effect of parameter addition or removal. **A.** Truncated SCRIT vectors containing only data collected in glu+ conditions demonstrate hierarchical clustering where direct mitochondrial toxicants oligomycin, antimicynA, rotenone, and valinomacin separated into different clusters. **B.** Six parameters were used for the statin SCRIT vector formation to detect changes in cell response induced by individual statins. Glu+ - glucose-containing medium, Glu− - glucose-free medium, 1 – TMRM integral, 2 – Hoechst33342 mean 3 – viability, 4 – TMRM maximum pixel, 5 – nuclear area, 6 – nuclear circularity. Arrow points to the TMRM max pixel dose response part of the SCRIT vector.

The demonstration of careful selection of image features (cell parameters), as well as normalization and vectorization of multifactorial responses, enabled clustering of tested compounds into groups associated with mechanism(s) of mitochondrial perturbation. The constructed SCRIT vectors were used to build hierarchical response classes, and were employed to perform MDS enabling visualization of compound classes in the reduced space of biological responses. For example, direct mitochondrial toxicants like rotenone and antimycin A were all attributed to the well-defined separated cluster (group I) when differential dose response and 60-dimensional SCRIT vectors were utilized. For comparison, a similar analysis was performed using SCRIT vectors that included only data collected in glucose-rich conditions, without the effect of glucose depletion (Figure 4A). The well isolated cluster of direct mitochondrial toxicants was lost and compounds like antimycin A, oligomycin, valinomycin, and rotenone were separated into different clusters (Figure 3A) when unifactorial data were processed..

Compounds like ionomycin formed another well-defined cluster (group II) that combines compounds whose toxicities are not compensated by glucose medium. This group may either activate mitochondria-independent death pathways with accompanying epi-phenomenal MMP decrease or induce mitochondria permeability transition independently of glycolysis. Group III are compounds that impact mitochondria function only under conditions of glucose limitation. Indeed, glucose withdrawal resulted in the decrease of cell viability accompanied by MMP decrease. Compounds like tacrine and nilutamide also display such behavior while clustered to group IV.

A cluster of compounds showing minimal or non-toxic responses in both conditions are largely approved drugs in group IV. A potential caveat is that these compounds may require metabolic activation by specific enzymes that are not expressed in HepG2 cultures. Conversely, some of these compounds can be enzymatically deactivated by HepG2 cells. These pharmacokinetic features are not included in the model at this point. A future application of the SCRIT-based differential response screen could be comparison of the differential effects of compounds with and without activation of specific cytochrome P450 (CYP) enzymes. A second cell model selected for known enzymatic expression patterns would be a likely approach to further distinguish the drugs in this cluster.

FCCP represents compounds with the potential to uncouple mitochondria oxidative-phosphorylation from ATP synthesis, and the cell could potentially survive through glycolysis (group V). The distinction from group II is based upon change of viability with no effect on TMRM. FCCP is largely not associated with a general toxic effect on cell viability and argues that it represents a unique mechanistic class [20].

Other image features can be extracted from the collected data and added to analysis by increasing the length of the SCRIT vector. An advantage is that more detailed information on fine differences between drugs within a therapeutic class can be derived. For example, out of three tested statins (HMG-Co A reductase inhibitors), simvastatin was clustered into group III based upon glucose-independent phenotypic responses at high concentration while pravastatin and fluvastatin were clustered together into group IV. When three more parameters were added to the SCRIT vectors, fine differences in cell responses to pravastatin and fluvastatin were revealed (Figure 4B). Even though the increase in TMRM intensity induced by fluvastatin over pravastatin is noticeable by visual examination of images (Figure 4B, top panel), TMRM peripheral integral signal intensity (Parameter 1 on Figure 4B) did not change. On the other hand, the maximal value of intensities associated with TMRM (“max pixel” parameter in iCys) increased dramatically in cases of simvastatin and fluvastatin but not pravastatin. TMRM maximal pixel parameter reflect MMP increases might occur due to alterations of cytoplasmic morphology from decreased cell adhesion and spreading, leading to higher cytoplasmic density in proximity to the nucleus. When TMRM maximum pixel intensity was analyzed, the increase in fluvastatin-treated over pravastatin-treated cells was also apparent (Parameter 4 on Figure 4B). The differential toxicities of statins in a variety of assays has been demonstrated; the more hydrophilic pravastatin is minimal, while lipophilic statins, including simvastatin and fluvastatin, are known to reduce hepatocyte viability and impair skeletal muscle mitochondria [25], [26], [27], [28]. Using these additional features reveals that fluvastatin is closer to simvastatin then to pravastatin (Figure 4B) in the space of biological responses.

**Figure 5:**
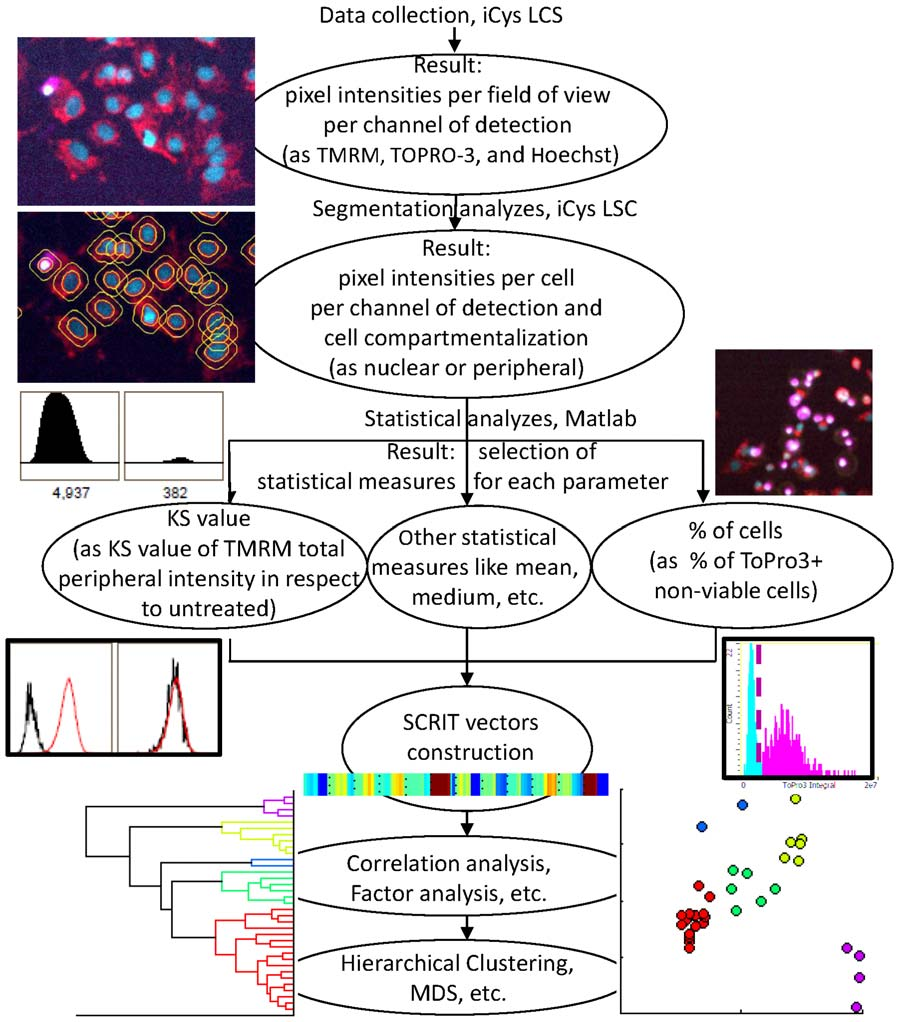
Schematic demonstration of high content screen and analysis. The screen algorithm is schematically represented the steps from cell culturing to data collection and comprehensive data analysis. HepG2 cells were seeded on 96-well plates either in regular culturing medium containing 5.5 mM glucose (glu+) or in glucose-depleted (glu−) medium containing10 mM galactose instead. Compounds were incubated for 24 h. Multiple controls per plate allowed correction of systematic well-to-well changes during data analysis. After incubation for 45 min with cell markers Hoechst33342, TMRM, and TO-PRO-3, plates were analyzed on the iCys. Six fields of view, each 500 µm×369 µm, were scanned per well with a single-pixel resolution of 0.5 µm. The initial image analysis of nuclear segmentation and determination of primary peripheral interval (PI) for the cytoplasmic signal was performed using iCys Cytometric Analysis software. Nuclear segmentation was performed according to the empirically-defined Hoechst33342 intensity threshold value and maintained throughout the screening. Further data reduction included KS value calculation for each parameter derived from TMRM and Hoechst33342 markers and per cent of viable cells derived from TO-PRO-3 using the empirical gating threshold value for TO-PRO-3 intensity. All parameters were assembled in a SCRIT vector and subjected to dissimilarity metrics calculation with consecutive correlation and MDS analysis.

This example of defining a quantitative metric for differential mitochondrial toxicity using a semi-automated data analysis provides a new tool set for assessing chemical effects on biological systems. The schematic outline for screening and analysis using the overall approach can be generalized by the work-flow shown in Figure 5. As presented, the example of mp-HCS using differential phenotypes highlights a novel experimental and computational approach for describing drug effects based upon analysis of dissimilarities between normalized response vectors. By computing a pair-wise distances between compounds on the basis of their SCRIT, our analysis possesses a capacity to associate unknown compounds with known mechanisms of action. The current unsupervised classification schema assumes realistic changes of the values of selected parameters using dose and glucose content as factors. Further subdivision of classes could be created by incorporating additional parameters (features) or conditions (factors). Integration of additional features is shown to be useful in distinguishing among drugs of a similar therapeutic class. Moving this process forward toward the challenge of predictive *in vitro* models appears increasingly feasible. These experimental and computational approaches will serve as starting points to address methodologies for drug dosing and/or development of safer practices with pharmacotherapies.

## Materials and Methods

### Cell culture and reagents

All fluorescent biomarkers and cell culture reagents were obtained from Invitrogen (Carlsbad, CA), D-galactose was from Sigma. Chemicals were obtained from different sources as listed in Table S1. The HepG2 human hepatocyte cell line was purchased from ATCC (Manassas, VA) and cultures were maintained in a humidified incubator under 5% CO_2_ at 37°C in MEM supplemented with 10%FBS, l-Glutamax, and sodium pyruvate. After attaining 90% confluence, cells were sub-cultured at 1∶3 ratios following trypsinization with 0.25% trypsin/EDTA solution. For glucose-free experiments, cells were maintained in glucose-free MEM, supplemented with 10% glucose-free FBS, l-Glutamax, sodium pyruvate, and 10 mM galactose. HepG2 cells were maintained at passage number below 20.

### Assay procedure

One day before the start of an experiment, cells were suspended by trypsinization and immediately robotically seeded at a density of 4.0×10^5^ cells/ml in 96-well collagen I–coated microplates (BD Biosciences) using a Biomek 3000 Laboratory Automation Workstation (Beckman Coulter) established in a tissue culture hood. Cells were allowed to adhere for 18–24 h before compound additions. For primary screening and secondary dose-response experiments, compounds at specific concentrations were formatted using a Biomek, added to plated cells, and incubated for 24 h at 37°C, 5% CO_2_.

### Live cell marker stains

Following the incubation period, addition of dye markers was performed with a staining procedure involving a cocktail of three cell markers for the imaging cytometry analyses. The final concentrations of dyes in each of the 96-wells were 125 nM TMRM, 133 nM TO-PRO-3, 1.5 µg/ml Hoechst33342, and 20 µM verapamil (see Methods S1). After incubation of the cell-containing plates for another 45 min at 37°C, 5% CO_2_, the cells were immediately analyzed.

### Data Collection

Ninety-six well plates were analyzed using an iCys Imaging Cytometer (Compucyte, Corp.) configured with three excitation lasers and four emission detector PMTs for blue, green, orange, and near-infrared. Six fields-of-view (500×368.6 µm each) were collected per well using a 20× objective at 0.5 µm resolution (0.5×0.48 µm pixel size). TMRM was excited by the 488 nm Ar laser and the emission recorded using PMT with 580/30 band-pass filter, Hoechst 33342 was excited by a 405 nm diode laser and emission recorded using PMT with 463/39 filter, and TO-PRO-3 was excited by a 633 nm He/Ne laser and emission recorded using PMT with 650 LP filter. The plate scanning protocol was set to collect row well data in a z-manner where one row is scanned after a row.

### Data analysis

Image segmentation was performed using the iCys Cytometric Analysis software (CompuCyte, Corp.). The protocol used single primary contour to identify cell events, and a peripheral contour to quantify cytoplasmic signal (Figure S1). The Hoechst33342 channel was used to find primary contours on a basis of adjacent pixels above the preset threshold value (2500 a.u.). The primary contours identify the presence of cells. The threshold value was set once and maintained for the entire screen. Low pass 5×5 smoothing filter and watershed procedure were applied to separate closely spaced or overlapping nuclei. Additionally, an area filter/discriminator was applied to eliminate clumps (max area 250 µm^2^) and cell debris (min 20 µm^2^). For the purpose of intensity integration, the primary contours were expanded by 4 pixels, and the peripheral contours were set as a 14 pixels-width ring. Unless otherwise stated, the parameter used for intensity of TMRM was peripheral integral signal. In cases of extended SCRIT, the TMRM maximal pixel intensity was used in addition. After image feature extraction, further statistical analysis of population distributions and graphical views were performed using Matlab 7.12.0 (The MathWorks) and OriginPro 8 software tools (OriginLab).

Testing of a 96-well plate with half of the wells treated with 75 µM FCCP (positive control) and half with 0.2% DMSO (negative control) was used for (1) definition of statistical measure of parameters, (2) well-to-well variability calculation, and (3) Z′ calculation for estimate of separation between positive and negative controls, and (4) robustness of SCRIT definition and computation of distances between SCRIT vectors.

1. KS distances were established as dissimilarity measures for TMRM- and Hoechst33342-based parameters, and percentage of live cells (viability factor) was established as a measure of TOPRO-3-derived parameter. TO-PRO-3 average intensity threshold values were 10^3.25^ a.u. of intensity with the exception of five plates that required threshold adjustments to 10^4^ a.u.
2. The selected statistical measures exhibited low level of intra-plate and inter-plate variability. For instance, maximum pixel TMRM KS distance between negative control and FCCP positive control samples had intra-plate CV of approximately 1.5, and inter-plate relative difference of approximately 15%.
3. A univariate control TMRM-based Z′ factor for each plate was calculated to illustrated separation between untreated samples and positive control in terms of the MMP parameter of SCRIT vector. In the context of this assay, the computed Z′ shows attainable separation in terms of TMRM peripheral integral between two samples. Since the goal of the described method is to perform semi-supervised classification, rather than binary separation of two classes (screen), the reported Z′ is interpreted only as a tool to assess system robustness, rather than an indicator of the overall quality of the assay. Therefore, the important factor is the reproducibility of the computed Z′, rather than the absolute value.
  KS distances between distributions of TMRM integral from pooled negative control (untreated) and relevant samples (wells) were used for the calculation. For each plate, its control Z′ was found according to the standard formula:
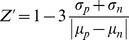
where *μ_p_* is the mean of KS distances between positive controls (FCCP treated samples) and the pooled negative control distribution; *μ_n_* is the mean of KS distances between each negative control and the pooled negative control distribution; *σ_p_* is the standard deviation of KS distances between positive controls and the pooled negative control, and *σ_n_* is the standard deviation of KS distances between individual negative controls and the pooled negative control distribution. The computed control Z′ values for individual plates were approximately 0.6–0.7. This value was stable and only minor fluctuations were noted between plates demonstrating the robustness of the assay conditions.
4. In order to estimate variability in the secondary assay, we computed average pair-wise distances between SCRIT vectors representing repeated measure of FCCP controls. For the 3-parametric SCRIT vectors constructed with TMRM peripheral integral, Hoechst33342 intensities and viability measured at a single drug concentration, the average distance was 0.15.

## Supporting Information

Figure S1 **The iCys data processing pipeline.**
(TIF)

Figure S2 **Effect of Verapamil (V) on TMRM fluorescence.** HepG2 were cultured on 96-well platesplate for 24 h before staining. Forty-eight samples (wells) were stained with dye mix (TMRM. Hoechst33342, and TO-PRO-3) without Verapamil (top, red border) and 48 samples with the same dye mix containing 20 µM Verapamil (bottom, blue border). **A.** Representative images (300×300 µm) of the TMRM channel (left) and merged fluorescent channels (TMRM - red, Hoechst 33342Hoechst33342 - blue, TO-PRO-3 - magenta) overlaid with the scatter channel (right) are shown. Note the homogenous staining of cells with TMRM in the presence of Verapamil and the heterogeneous staining without it. The arrow points to the morphologically normal cell showing no TMRM staining. **B.** TMRM maximal pixel intensity histograms for all analyzed cells are shown fitted with a Gaussian function to enable statistical analysis. Blue lines represent data collected from samples stained without Verapamil, red lines from those with Verapamil in the dye mix. The Table below the graph summarizes statistical parameters that describe the Gaussian distribution.
(TIF)

Figure S3 **Differential dose response and SCRIT vector construction.** HepG2 were incubated with different concentrations of compounds (0.001 µM to 100 µM) in glu+ or glu− conditions, stained with TMRM, Hoechst 33342, and TO-PRO-3, and analyzed by LSC. **A**. Graphs show dose responses for KS values of TMRM IF and VF of TO-PRO-3 for glu+ and glu− conditions for FCCP (black), ionomycin (red), rotenone (green), and antimycin A (blue). **B**. The same graphs are represented as signature cell responses to induced toxicity (SCRIT) vectors to demonstrate specific patterns of cell response to different compounds in different media. Cell viability is rescaled to the [−1, 1] range, where −1 reflects all dead cells and +1 - all alive, to match the range of KS distances. Four dose response series for each compound, representing four repeats, are plotted on the same graph in different colors to demonstrate assay robustness. **C**. 40-data-point SCRIT vectors that incorporate two parameters, 10 concentrations, and 2 medium conditions (glu+ and glu−) are represented in a color-coded format. The four repeats of each of four compounds area plotted in color representing the SCRIT vectors. Color scale is shown below.
(TIF)

Figure S4 **Schematic diagram of primary screening process.** Primary screening was performed using a single high concentration of the tested compounds (100 µM).). The cells were exposed to the compounds for 24 h in glu+ and glu− conditions. 76 compounds were tested on a 96-well plate; 4 FCCP-treated positive and 4 DMSO-treated negative controls were also included on the same plate. After 24 h of incubation cells were stained with TMRM, Hoechst 33342, and TO-PRO-3 as described in the Materials and Methods section. Representative fields of view collected with iCys show three merged channels. The experiment was performed in duplicate for both glu+ and glu− conditions. Seven parameters were further analyzed statistically. SCRIT vectors demonstrate the seven-parametric response to individual drugs in glu+ and glu− conditions (a total of 14 parameters/compound), where normalized KS distance between control and compound treated cells using 1 – TMRM peripheral integral 2 – TMRM maximum pixel, 3 – Hoechst 33342 mean, 4 – Hoechst 33342 integral, 5 – nuclear area, 6 – nuclear circularity, and 7 – the percentages of viability, from glu+ – (glucose-containing) and glu− – (glucose-free) medium. Computation of Euclidian distances was followed by visual examination of similarities between SCRIT vectors in order to select responsive compounds. These compounds were kept for a secondary screen. The compounds showing lack of activity in the primary screen were designated to group IV – non-toxic or inactive compounds. A change of more than 20% in any one of seven parameters and/or a change of cell morphology detected by visual analysis of the transmitted light image was considered as a marker of response. These compounds were kept for secondary screening.
(TIF)

Figure S5 **Types of anomalous excitation induced by several compounds.** Representative images (500×368 mm each) for non-treated controls and for cultures treated for 6 h with 20 µM doxorubicin, 20 µM tamoxifen, and 20 µM camptothecin, and for 24 h with 100 µM menadione are shown. Merges of three fluorescent channels (TMRM - red, Hoechst 33342 - blue, TO-PRO-3 - magenta) are shown in the left column and corresponding transmitted light images are shown on the right. Note the marked decrease of Hoechst 33342 staining after treatment with doxorubicin and tamoxifen. Doxorubicin and menadione autofluorescence masks TMRM at high concentrations. Incubation with camptothecin resulted in substantial autofluorescence background in the Hoechst 33342 channel, masking nuclear staining and interfering with segmentation. Lower but considerable Hoechst 33342–channel autofluorescence was also caused by lapatinib and dasatinib (data not shown).
(TIF)

Methods S1 **Additional detailed description are provided for automated mpHCS for mitochondrial toxicity protocol, data collection and analysis, TMRM staining optimization, multiparametric analysis.**
(DOCX)

Table S1 **A distance matrix between SCRIT vector responses.** Pair-wise distances are the basis for hierarchical clustering and subsequent multi-dimensional scaling (MDS) analysis. A small, low pair-wise distance represents a high level of similarity. Individual samples are shown, including multiple repeats of each compound. Small values of pair-wise distances between repeats also demonstrate low variability in the experiment. This is a truncated table provided for demonstration and the full matrix is available upon request and the values have been round off.
(XLSX)

Table S2 **Compounds used for phenotypic toxicity characterization.** Complete list of compounds used in the experiment with their alternative names are shown. Primary screening results with respect to toxicity (“toxicity” or “no toxicity”) and incompatibility with automatic fluorescence analysis (“outlier”) are shown. Results of secondary screening are shown as toxicity group number, where I - direct mitochondrial toxins, II - glu-independent toxins, III - glu-dependent toxins, IV - non-toxic or inactive compounds, and V - uncoupling compounds such as FCCP.
(XLSX)
