# Supplementary figures and images for: Differential Mitochondrial Toxicity Screening and Multi-Parametric Data Analysis
Source: PLoS One. 2012 Oct 15;7(10):e45226. doi: 10.1371/journal.pone.0045226 (PMC3471932; doi:10.1371/journal.pone.0045226)

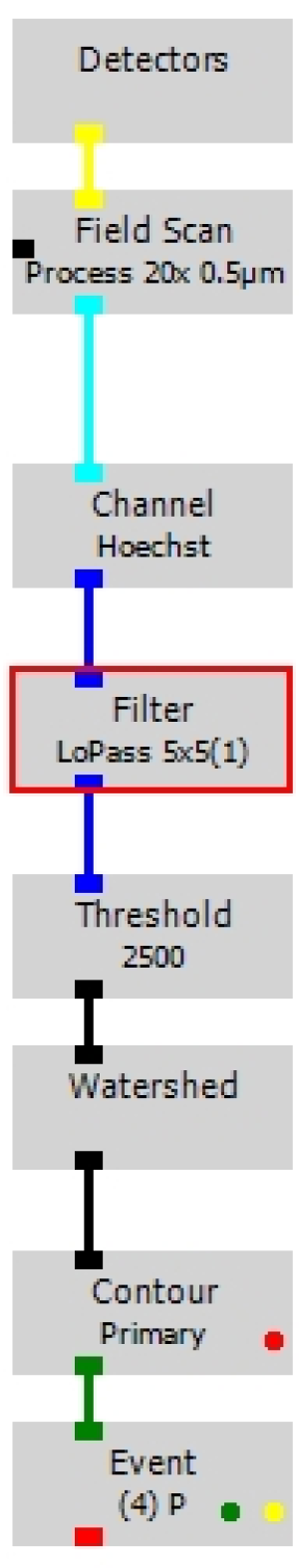

Supplement: Figure S1 — The iCys data processing pipeline. (TIF) [file pone.0045226.s001.tif]

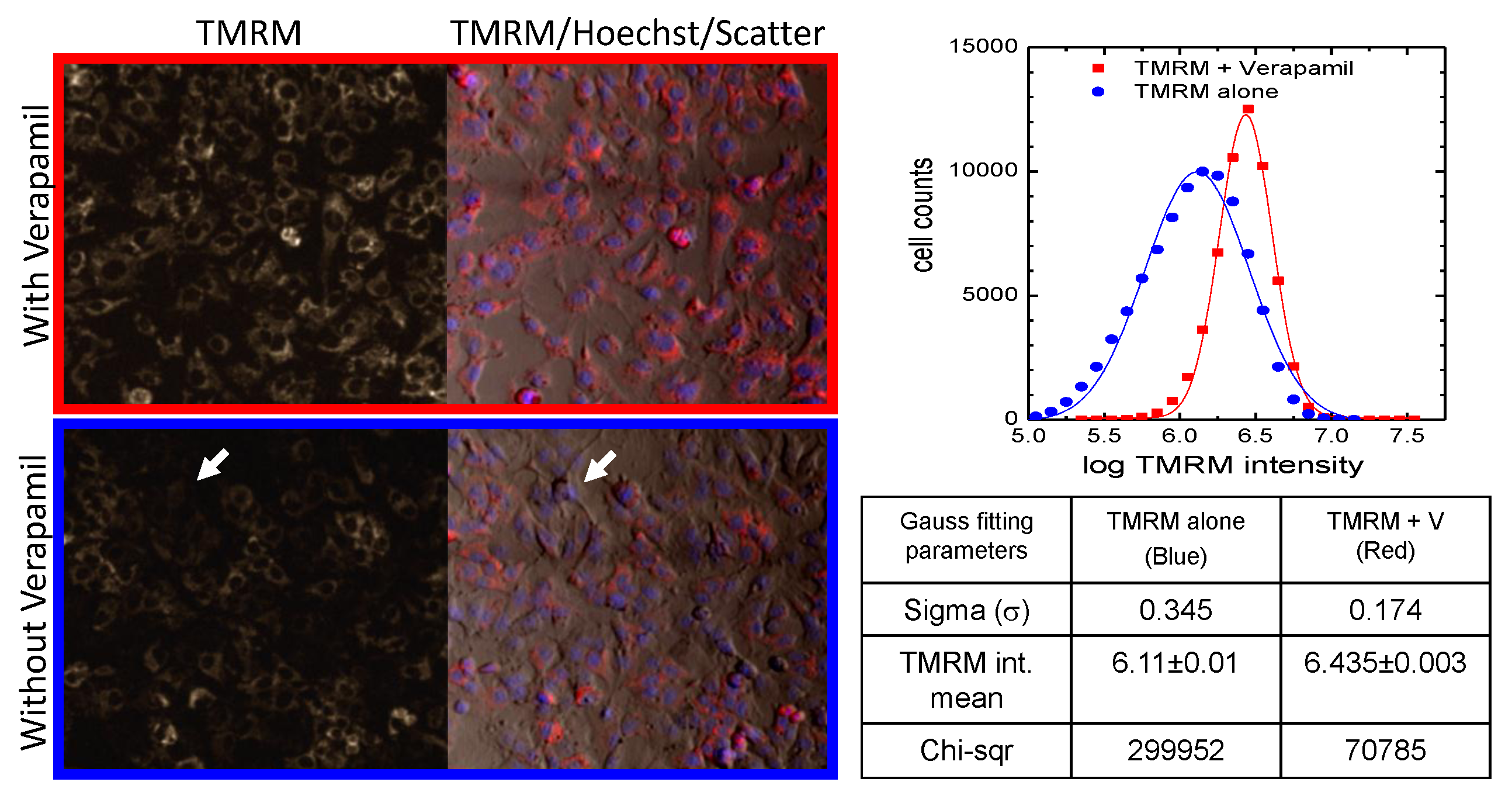

Supplement: Figure S2 — Effect of Verapamil (V) on TMRM fluorescence. HepG2 were cultured on 96-well platesplate for 24 h before staining. Forty-eight samples (wells) were stained with dye mix (TMRM. Hoechst33342, and TO-PRO-3) without Verapamil (top, red border) and 48 samples with the same dye mix containing 20 µM Verapamil (bottom, blue border). A. Representative images (300×300 µm) of the TMRM channel (left) and merged fluorescent channels (TMRM - red, Hoechst 33342Hoechst33342 - blue, TO-PRO-3 - magenta) overlaid with the scatter channel (right) are shown. Note the homogenous staining of cells with TMRM in the presence of Verapamil and the heterogeneous staining without it. The arrow points to the morphologically normal cell showing no TMRM staining. B. TMRM maximal pixel intensity histograms for all analyzed cells are shown fitted with a Gaussian function to enable statistical analysis. Blue lines represent data collected from samples stained without Verapamil, red lines from those with Verapamil in the dye mix. The Table below the graph summarizes statistical parameters that describe the Gaussian distribution. (TIF) [file pone.0045226.s002.tif]

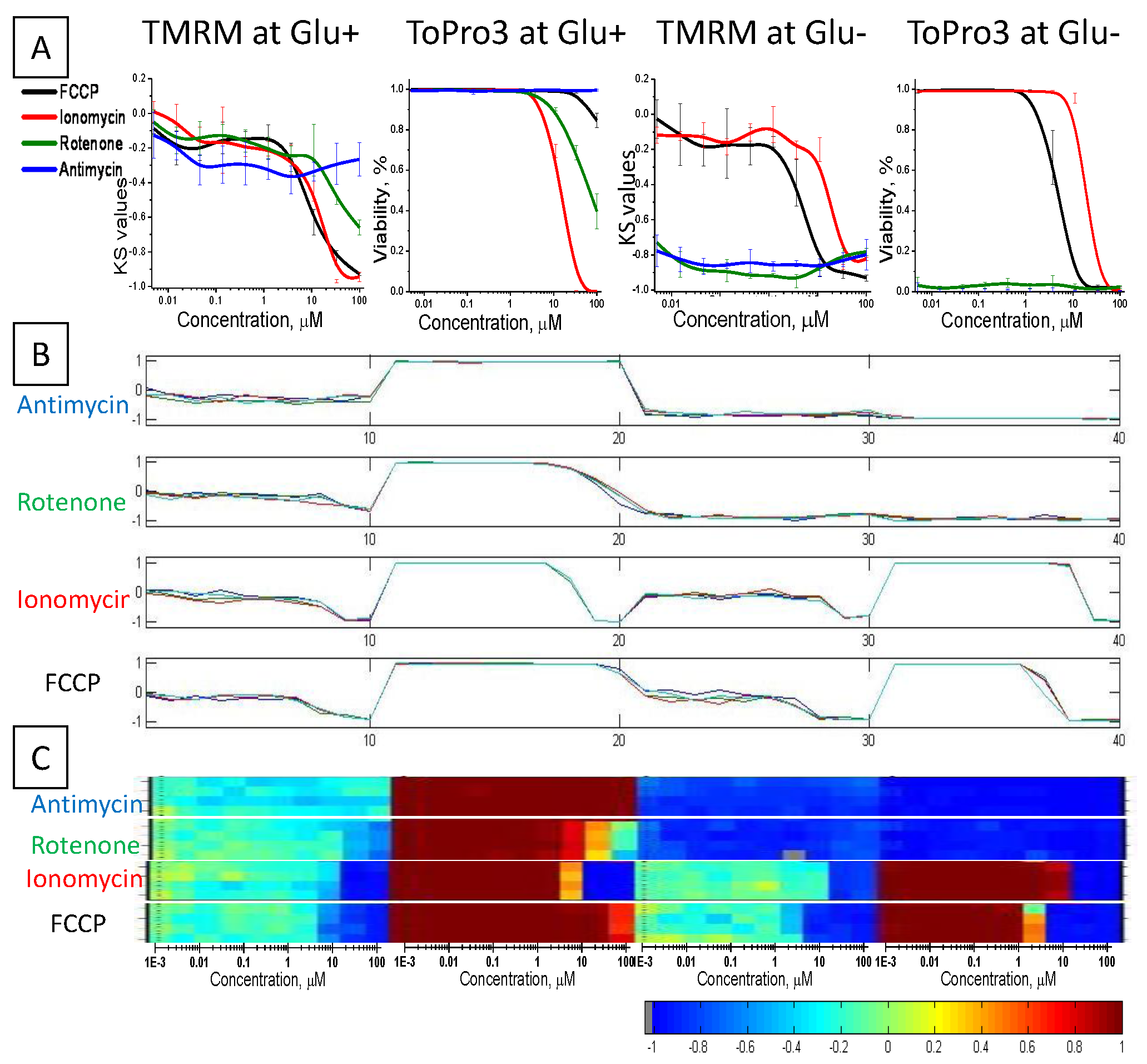

Supplement: Figure S3 — Differential dose response and SCRIT vector construction. HepG2 were incubated with different concentrations of compounds (0.001 µM to 100 µM) in glu+ or glu− conditions, stained with TMRM, Hoechst 33342, and TO-PRO-3, and analyzed by LSC. A. Graphs show dose responses for KS values of TMRM IF and VF of TO-PRO-3 for glu+ and glu− conditions for FCCP (black), ionomycin (red), rotenone (green), and antimycin A (blue). B. The same graphs are represented as signature cell responses to induced toxicity (SCRIT) vectors to demonstrate specific patterns of cell response to different compounds in different media. Cell viability is rescaled to the [−1, 1] range, where −1 reflects all dead cells and +1 - all alive, to match the range of KS distances. Four dose response series for each compound, representing four repeats, are plotted on the same graph in different colors to demonstrate assay robustness. C. 40-data-point SCRIT vectors that incorporate two parameters, 10 concentrations, and 2 medium conditions (glu+ and glu−) are represented in a color-coded format. The four repeats of each of four compounds area plotted in color representing the SCRIT vectors. Color scale is shown below. (TIF) [file pone.0045226.s003.tif]

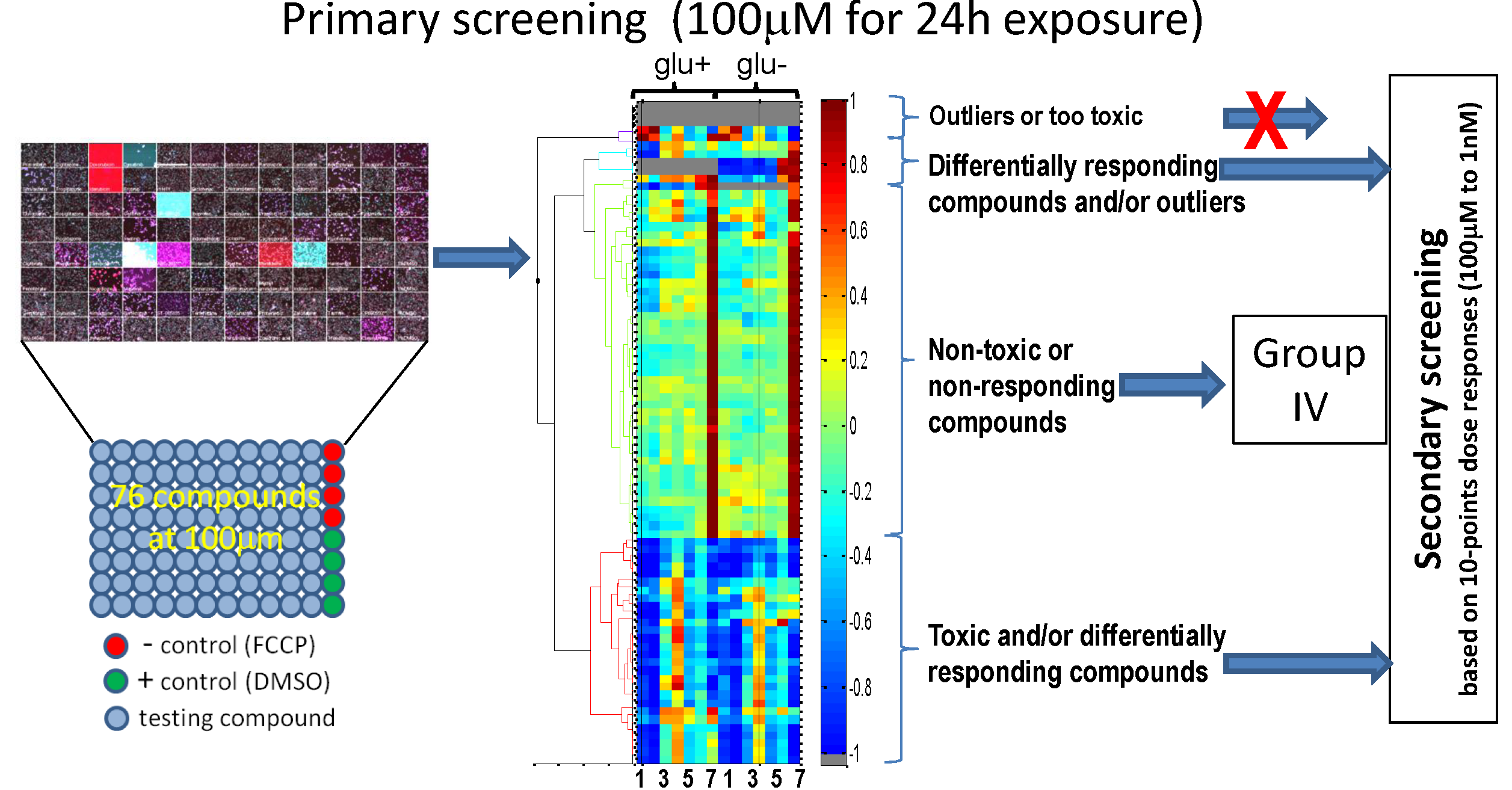

Supplement: Figure S4 — Schematic diagram of primary screening process. Primary screening was performed using a single high concentration of the tested compounds (100 µM).). The cells were exposed to the compounds for 24 h in glu+ and glu− conditions. 76 compounds were tested on a 96-well plate; 4 FCCP-treated positive and 4 DMSO-treated negative controls were also included on the same plate. After 24 h of incubation cells were stained with TMRM, Hoechst 33342, and TO-PRO-3 as described in the Materials and Methods section. Representative fields of view collected with iCys show three merged channels. The experiment was performed in duplicate for both glu+ and glu− conditions. Seven parameters were further analyzed statistically. SCRIT vectors demonstrate the seven-parametric response to individual drugs in glu+ and glu− conditions (a total of 14 parameters/compound), where normalized KS distance between control and compound treated cells using 1 – TMRM peripheral integral 2 – TMRM maximum pixel, 3 – Hoechst 33342 mean, 4 – Hoechst 33342 integral, 5 – nuclear area, 6 – nuclear circularity, and 7 – the percentages of viability, from glu+ – (glucose-containing) and glu− – (glucose-free) medium. Computation of Euclidian distances was followed by visual examination of similarities between SCRIT vectors in order to select responsive compounds. These compounds were kept for a secondary screen. The compounds showing lack of activity in the primary screen were designated to group IV – non-toxic or inactive compounds. A change of more than 20% in any one of seven parameters and/or a change of cell morphology detected by visual analysis of the transmitted light image was considered as a marker of response. These compounds were kept for secondary screening. (TIF) [file pone.0045226.s004.tif]

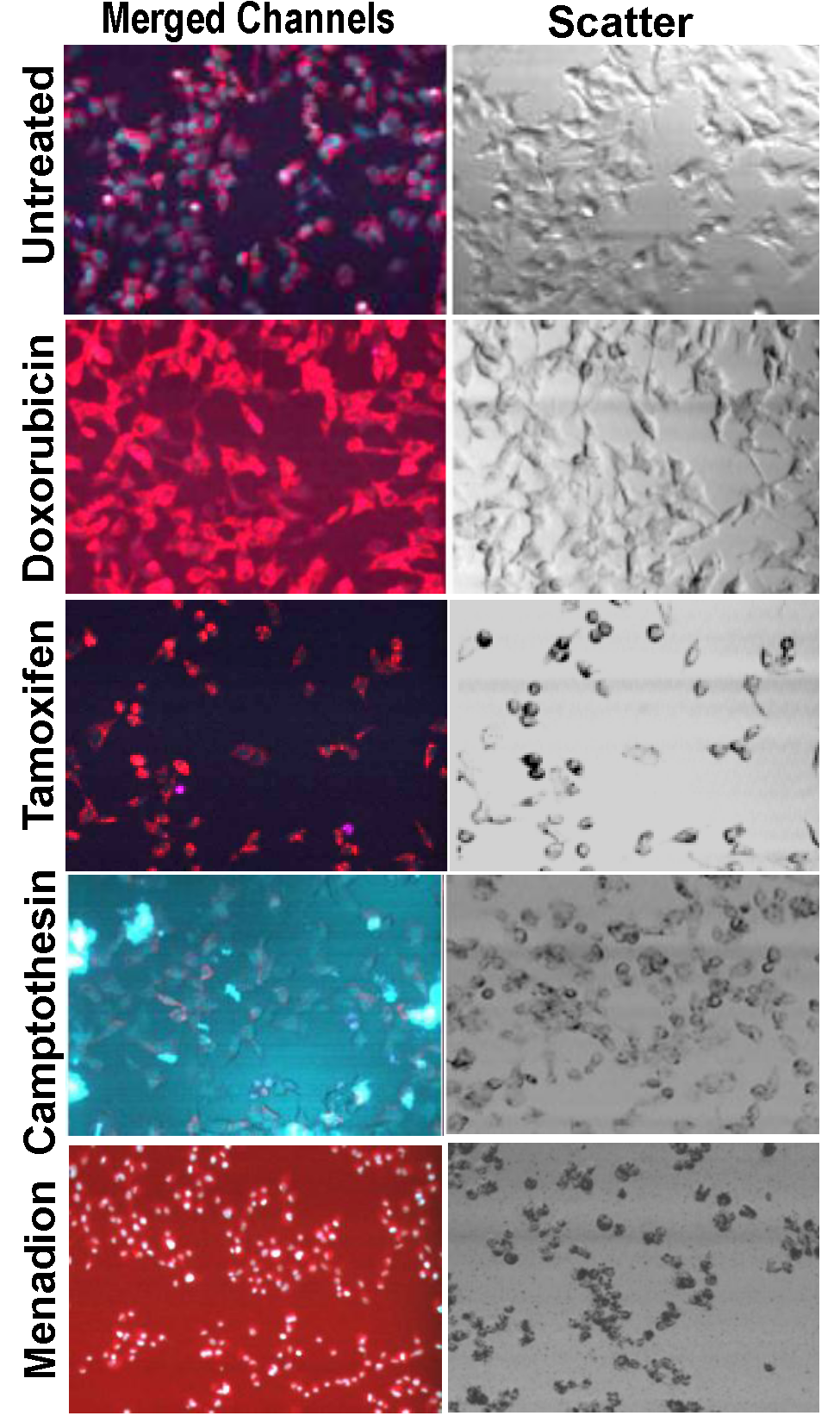

Supplement: Figure S5 — Types of anomalous excitation induced by several compounds. Representative images (500×368 mm each) for non-treated controls and for cultures treated for 6 h with 20 µM doxorubicin, 20 µM tamoxifen, and 20 µM camptothecin, and for 24 h with 100 µM menadione are shown. Merges of three fluorescent channels (TMRM - red, Hoechst 33342 - blue, TO-PRO-3 - magenta) are shown in the left column and corresponding transmitted light images are shown on the right. Note the marked decrease of Hoechst 33342 staining after treatment with doxorubicin and tamoxifen. Doxorubicin and menadione autofluorescence masks TMRM at high concentrations. Incubation with camptothecin resulted in substantial autofluorescence background in the Hoechst 33342 channel, masking nuclear staining and interfering with segmentation. Lower but considerable Hoechst 33342–channel autofluorescence was also caused by lapatinib and dasatinib (data not shown). (TIF) [file pone.0045226.s005.tif]
