# Supplementary material for: Differential Mitochondrial Toxicity Screening and Multi-Parametric Data Analysis
Source: PLoS One. 2012 Oct 15;7(10):e45226. doi: 10.1371/journal.pone.0045226 (PMC3471932; doi:10.1371/journal.pone.0045226)
Supplement: Methods S1 — Additional detailed description are provided for automated mpHCS for mitochondrial toxicity protocol, data collection and analysis, TMRM staining optimization, multiparametric analysis. (DOCX) [file pone.0045226.s006.docx]

# Supplemental METHODS

## Detailed description of automated mpHCS for mitochondrial toxicity protocol

The assay was developed as a high-content automated semi-supervised classification procedure conducted in 96-well microplates. The total volume of 120 μl per well was dispensed sequentially using a Biomek 3000 Automated Workstation (Beckman Coulter). Cells cultures were prepared as follows: 1) 50 μl of 4×10^5^ cells/ml HepG2 cells were seeded in either glucose-containing (glu+) or glucose-free (glu-) medium 18-24 h before compound addition. Glu+ medium: MEM (Invitrogen, #51200), 10% FBS (Invitrogen, #26400), l-Glutamax (Invitrogen, #35050), sodium pyruvate (Invitrogen, #11360), antibiotics (Invitrogen, #15140); Glu- medium: MEM (Invitrogen, #51200), 10 mM d-galactose (Sigma, G5388), 10% FBS (Invitrogen, #26400), l-Glutamax (Invitrogen, #35050), sodium pyruvate (Invitrogen, #11360), antibiotics (Invitrogen, #15140); 2) 50 μl test compound was added from a master compound plate. The master compound plate was prepared using a Biomek Automation Workstation. Specifically for 10-point dose- response secondary screening, 10 (1:3) dilutions of 4 different compounds (2 replicates per compound) were on the same plate along with the first column of FCCP at 150 μM and the last column of no-treatment control containing 0.2% DMSO. The final top concentration of compounds on the master compound plate varied from 200 μM to 4 nM; 3) cells were incubated with compounds for 24 hours at 37^o^C and 5% CO_2_; 4) 10 μl concentrated dye mix I (1.375 μM TMRM, 1.467 μM TO-PRO-3, 16.5 µg/ml Hoechst 33342, and 220 µM verapamil in glu+ or glu- MEM, depending on the experiment) was added using the Biomek FX; 5) plate was incubated with dyes for 45 min at 37^o^C and 5%CO_2_; 6) the plate was immediately analyzed using the iCys imaging cytometer (CompuCyte, Corp. Cambridge, MA).

## iCys data collection and analysis

The following parameters and data processing pipeline were developed and maintained for the screening (Figure S1). Six fields-of-view were collected using a 20x objective at 0.5 micron resolution. TMRM was excited by the 488 nm Ar laser and emission recorded using PMT with 580/30 band-pass filter, Hoechst 33342 was excited by a 405 nm diode laser and emission recorded using PMT with 463/39 filter, and TO-PRO-3 was excited by a 633 nm He/Ne laser and emission recorded using PMT with 650 LP filter. **Field scan:** objective lens 20x, step size 0.5µm, field size 500x368.6 µm (1000x768pixels), pixel size 0.5x0.48 µm. **Primary detection channel:** Hoechst33342 was selected for primary contour definition. **Filters:** a 5x5 low- pass smoothing filter was used prior to segmentation. **Segmentation:** the value of 2500 A.U. was established empirically as optimal and maintained for the entire study in order to perform binarization of the primary contours. **Separation of contours:** an iCys watershed procedure involving a series of erosion and dilation steps was used to separate closely spaced or overlapping contours. The settings for the iCys watershed: sensitivity 0.97, ridge length 3 pixels min, 8 pixels max. **Size discrimination:** binarized nuclei with minimal size of 20 µm^2^ and maximal size of 250 µm^2^ were employed to define cell location. **Integration of fluorescence signals:** subsequently to binarization, integration contours were expanded by 4 pixels. The iCys dynamic background setting was kept off. Peripheral contours were 14 pixels wide. There was no additional separation between the primary and the peripheral integration contours.

## TMRM staining optimization

TMRM is known to be a substrate for multi-drug resistance (MDR) transporters. especially for the p-glycoprotein (*1-5*). MDR transporters are membrane channels belonging to the ATP-binding cassette (ABC) transporters family that function as small- molecules efflux pumps. There are at least three reasons to take MDR transport into account. First, populations of cultured cells could be heterogeneous for the activity of MDR transporters and hence TMRM loading could vary from cell to cell. Second, testing compounds may affect intensity of TMRM indirectly through inhibition of MDR transport. And finally, since the activity of MDR transporters is ATP-dependent, the presence or absence of glucose in the culturing medium could introduce variability when glucose-dependency is a factor. Thus, we incorporated the MDR p-glucoprotein (MDR1) transporter inhibitor Verapamil in the TMRM staining protocol to normalize the cell loading with TMRM, and to inhibit MMP-independent changes in the TMRM signal. We also analyzed other MDR inhibitors and found that Probenecid, which inhibits the MDR-associated protein MRP1, had no effect on TMRM loading, while Cyclosporin A resulted in a dramatic increase of fluorescence due to TMRM. Use of Cyclosporin A in the mitochondrial toxicity assay is objectionable due owing to its well-accepted functional inhibition of the mitochondrial transition pore (MTP).

The effect of Verapamil is shown in Figure S2. Forty-eight wells of a 96-well plate with cultured HepG2 cells were stained with the regular biomarkers mix (Hoechst 33342, TMRM, TO-PRO-3) without Verapamil, and 48 wells on the same plate were stained with the biomarkers mix containing 20 μM Verapamil. We found no difference between samples with or without Verapamil in Hoechst 33342 and TO-PRO-3 channels, while TMRM staining showed that addition of Verapamil made the population more homogeneous (standard deviation was reduced by a factor of 2).

## Multiparametric analysis

Seven parameters were analyzed for cell-response characterization. For the TMRM channel, integral fluorescence (IF) and max pixel fluorescence (MF) intensity values were used for the 14 -μm ring around the cell nucleus imitating the peripheral cytoplasmic area of a cell. The IF and MF of each cell in the field of view were used for the sample-population response assessment. For the Hoechst 33342 channel four parameters were extracted: nuclear area, circularity, Hoechst 33342 average fluorescence intensity (AF), and Hoechst 33342 integral fluorescence intensity (IF). For the TO-PRO-3 channel, average nuclear intensity was used (AF).

Comprehensive statistical analysis of the parameters distribution was performed using Origin 8 Pro (OriginLab Corporation, Northampton, MA) and Matlab 7.7.0 (The MathWork, Natick, MA, USA). Statistical measures of dissimilarity were defined for each parameter individually based on analysis of each distribution. For the TO-PRO-3 channel we calculated a cell viability factor (VF) as the percent of TO-PRO-3-negative (alive) cells, using TO-PRO-3 gating values, according to the formula VF=1-N_test-dead_/N_test-all_, where N_test-dead_ is the number of TO-PRO-3-positive cells in the tested sample, and N_test-all_ is the total number of cells in the tested sample based on Hoechst 33342 data. Kolmogorov-Smirnov (KS) distance was used to compare non Gaussian distributions ([*6*](#_ENREF_6)). KS distances between a distribution of cells exposed to a compound and a distribution of negative control (DMSO only) was computed using the formula

$$\text{KS}_{\left(\text{comp, control}\right)}=\sup\left|F_{\text{comp}}-F_{\text{cont}}\right|\cdot\mathrm{sgn}\left(F_{\text{comp}}-F_{\text{cont}}\right)$$

where *F_comp_* and *F_cont_* are the cumulative distribution functions of compound-treated and DMSO-treated (control) samples respectively. The sign of KS distance reflects the direction of the change in relation to the negative control (DMSO). For example: TMRM IF KS < 0 reflects decrease of TMRM (drop of MMP) and KS distance value > 0 reflects increase of TMRM (MMP hyperpolarization). KS values were calculated between each sample distribution and the corresponding negative control distribution for each compound, dilution, and parameter of interest. The variability of KS values depended on control-well selection. Consequently, Z΄ for TMRM MF computed between FCCP-treated and untreated samples was improved from 0.66 to 0.7 when negative controls were taken separately for each row. Use of separate control wells for each row lowers the impact of gradual changes in sample conditions during the 2.5 h iCys scan at room temperature. Thus, in order to lower variability, separate negative control samples were used for computing dissimilarity values building the response vectors.

KS distances were used further for response vector construction. VF was rescaled from -1 (all dead) to +1 (all alive) to match the range for signed KS distances. The KS values for TMRM IF, TMRM MF, Hoechst 33342 area, Hoechst 33342 circularity, Hoechst 33342 AV, Hoechst 33342 IF, and VF for TO-PRO-3 AF were calculated for each compound at every concentration during secondary screening in glu- and glu+ conditions (matrix size 152×140, data available). A single vector that characterizes comprehensive specialized cell response to induced toxicity (SCRIT vector) was generated for each drug by vectorizing the multifactorial dataset containing all the parameters in 10 concentrations and two media conditions (glu+ and glu-). Thus, up to 140-entry long SCRIT vectors were generated for every repeat of the tested compounds; examples of 40-entry long SCRIT vectors constructed using 2 parameters (KS values of TMRM IF and VF of TO-PRO-3) are shown in Figure S2. These vectors represent data from 10-data point dose response, at glu+ and glu- conditions for each of four known mitochondrial toxins, FCCP (black), ionomycin (red), rotenone (green), and antimycin A (blue). Individual dose responses (Figure S2 A, mean and std) combined dose responses of parameters, doses, and conditions (Figure S2 B, individual repeats shown), and color-coded SCRITs (Figure S2 C) are presented.

Further data reduction involved calculation of Pearson distances (1-correlation) between SCRIT vectors. The raw distance matrix of dissimilarity between SCRIT vectors built based on 10-point dose responses of 3 parameters (TMRM IF, Hoechst33342 IF, TO-PRO-3 AF) at 2 glucose-containing conditions is demonstrated in Table S1 (Truncated at 45 lines, full version is available). Complete-linkage hierarchical clustering of SCRITs (including repeated compounds as independent vectors) was performed in order to group compounds with similar SCRITs. When replicates of the same compound did not cluster together and had a distance > 0.1, such compounds were removed from final clustering results and recommended for separate study. Five broad-spectrum groups based on compounds differential mitochondrial involvement of compounds and toxicity identified in Table S2.

## References

1. Neyfakh, A. A. (1988) Use of fluorescent dyes as molecular probes for the study of multidrug resistance, *Experimental Cell Research* *174*, 168-176.

2. Litman, T., Zeuthen, T., Skovsgaard, T., and Stein, W. D. (1997) Structure-activity relationships of P-glycoprotein interacting drugs: kinetic characterization of their effects on ATPase activity, *Biochimica et Biophysica Acta (BBA) - Molecular Basis of Disease* *1361*, 159-168.

3. Eytan, G. D., Regev, R., Oren, G., Hurwitz, C. D., and Assaraf, Y. G. (1997) Efficiency of p-glycoprotein–mediated exclusion of rhodamine dyes from multidrug-resistant cells is determined by their passive transmembrane movement rate, *European Journal of Biochemistry* *248*, 104-112.

4. Bernardi, P., Scorrano, L., Colonna, R., Petronilli, V., and Di Lisa, F. (1999) Mitochondria and cell death, *European Journal of Biochemistry* *264*, 687-701.

5. Irwin WA, J. D., Antolovic R. (2008) Biomarkers for Drug Discovery: Important Aspects of in vitro Assay Design for HTS and HCS Bioassays, *Croatica Chemica Acta* *81*, 23-30.

6. Feng, Y., Mitchison, T. J., Bender, A., Young, D. W., and Tallarico, J. A. (2009) Multi-parameter phenotypic profiling: using cellular effects to characterize small-molecule compounds, *Nature Reviews Drug Discovery* *8*, 567-578.
